# Long-Term Prognosis of Quality of Life in Dogs Diagnosed With Mild to Moderate Elbow Dysplasia in Sweden

**DOI:** 10.3389/fvets.2020.572691

**Published:** 2020-11-03

**Authors:** Annika Bergström, Sofia Johard, Marcel H. Lee, Arianna Comin

**Affiliations:** ^1^Department of Clinical Sciences, Swedish University of Agricultural Sciences, Uppsala, Sweden; ^2^Anicura Stockholm Animal Hospital, Stockholm, Sweden; ^3^Södra Small Animal Hospital, Stockholm, Sweden; ^4^Department of Disease Control and Epidemiology, National Veterinary Institute, Uppsala, Sweden

**Keywords:** dog, elbow dysplasia, lameness, osteoarthritis, quality of life

## Abstract

**Objective:** The objective of this study was to increase knowledge regarding long-term prognosis of mild to moderate elbow dysplasia (ED) using a canine orthopedic index.

**Study Design:** Cross-sectional observational study.

**Sample Population:** Sixty dogs randomly selected from each of five different breeds and three ED groups: ED0 (control), ED1, and ED2, based on the Kennel Club's screening results. The total number of selected dogs was 900 (60^*^5^*^3).

**Methods:** Questionnaires were administered to owners by telephone interview. Bayesian network modeling was used to assess the relation between ED grade, treatment options, dog demographics, and quality-of-life indicators.

**Results:** Seven hundred sixty-five questionnaires were collected (85% response rate), of which 61 concerned dogs euthanized due to osteoarthritis. There was no direct association between ED grade and owner's perceived quality of life, but ED1 and ED2 dogs were more likely to receive veterinary care and subsequent NSAID treatment compared to ED0 dogs. A significant association was found between the occurrence of euthanasia due to orthopedic disease and ED scores 1 and 2 in the sample (*p* < 0.001).

**Conclusion:** The degree of osteoarthritis was not directly associated with the canine orthopedic index, except for ED2 and lameness score. It can be speculated that owners who paid closer attention to orthopedic symptoms and perceived them as impairing their dogs' lives were also more likely to seek veterinary care and get treatment, irrespective of the ED grading.

**Impact:** ED1-graded dogs had a lower risk than might be expected to develop visible clinical symptoms and showed a similar quality of life as dogs with ED0. ED2-graded dogs were more likely than ED0-graded dogs to have their lives impaired by lameness, according to the owners' perception.

## Introduction

Lameness is one of the most common reasons for seeking veterinary care, and elbow dysplasia (ED) is the most common cause of forelimb lameness in young large- and giant-breed dogs (1). ED is a collective term describing several different developmental elbow conditions including medial coronoid disease, radioulnar incongruity, ununited anconeal process, and osteochondrosis (2–7).

Many reports have been published regarding surgical and medical treatment for different ED conditions in dogs (1, 8–12). However, despite surgical or medical intervention, ED will inevitably result in progressive osteoarthritis (OA) in the long term, leading to pain and loss of function of the joint (13).

Like many countries, the Swedish Kennel Club grading system for ED is a quantitative radiographic evaluation by a specialist of OA in the elbow joint based on the International Elbow Working Group (IEWG) criteria. Young mature dogs from breeds at risk of developing ED are radiographically evaluated at 12 months or older in one flexed lateral projection of the elbow. The aim of the screening is to register any signs of OA, without focusing on the underlying cause of the OA (14).

The resulting ED score categorizes dogs into one of four groups based on the severity of their ED findings: ED0 for a normal joint, ED1 for mild, ED2 for moderate, and ED3 for severe arthrosis (Table 1) (15, 16). To the author's knowledge, there are currently no studies relating the ED screening score to a dog's long-term prognosis and quality of life.

**Table 1:** Elbow scoring modified from the International Elbow Working Group (IEWG) 2012 (15).

	**Elbow dysplasia scoring**	**Radiographic findings**
0	Normal elbow joint	Normal elbow joint, no evidence of sclerosis or arthrosis.
1	Mild arthrosis	Presence of osteophytes <2 mm high, sclerosis of the base of the coronoid processes, trabecular pattern still visible.
2	Moderate arthrosis	Presence of osteophytes of 2–5 mm high. Obvious sclerosis (no trabecular pattern) of the base of the coronoid processes, step of 3–5 mm between radius and ulna. Indirect signs of primary lesion (UAP, FCP/coronoid disease, OCD).
3	Severe arthrosis	Presence of osteophytes of >5 mm high. Step of >5 mm between radius and ulna (obvious INC). Obvious presence of a primary lesion (UAP, FCP, OCD).^*^

*UAP, ununited anconeal process; FCP, fragmented processus coronoideus; OCD, osteochondrosis dissicans; INC, incongruency*.

* *Radiographically or surgically confirmed and reported to the Kennel Club*.

The gold standard for lameness measurement is gait analysis (17), which is costly and time-consuming. To assess a larger population of dogs for lameness (with or without chronic OA), owner questionnaires can be used as an alternative to clinical assessment and objective measurements (18–23). Several validated owner questionnaires evaluating OA in dogs exist, including the Canine Orthopedic Index (COI), Liverpool Osteoarthritis in Dogs (LOAD), the canine brief pain inventory (CBPI), and the Helsinki chronic pain index (HCPI) (20, 21, 23–26).

The purpose of this study was to evaluate long-term owner-assessed quality of life, using COI and other owner questions, in dogs screened for ED. We hypothesized that the ED-positive dogs (ED1 or ED2) would be reported as suffering more from lameness and receive more veterinary care, as well as have a worse owner-assessed quality of life and COI scores compared to ED-negative dogs (ED0).

## Methods

### Animal Selection

Five breeds commonly diagnosed with ED by the Swedish Kennel Club were included: American Staffordshire Bullterrier, Bernese mountain dog, German Shepherd, Labrador Retriever, and Rottweiler.

Initially, 60 dogs from each breed were randomly selected by computer from the Swedish Kennel Club database for each of the following ED groups—ED0, ED1, and ED2—based on a radiographic evaluation that occurred within a 4-year period from January 2011 and January 2015 (Table 1). A fourth group (ED3) was considered but later removed from the study due to the small number of cases. Also, surgically treated dogs (due to ED) will always be categorized as ED3 by the Kennel Club regardless of any radiographic OA scoring.

All owners of selected dogs were sent a letter in the mail informing them about the study and inviting them to a telephone interview. Dogs whose owners did not respond or were unwilling to participate were replaced by new randomly selected dogs from the same ED group. Dogs that were euthanized for reasons other than orthopedic disease or that were sold were excluded. All interviews were performed in 2017.

### Questionnaire

The interview included three parts. The first collected demographic information on the dog: breed, age, gender, registration number, date of ED radiography, and ED grade and hip dysplasia grade. The second part asked about the occurrence of lameness in the past month and veterinary treatment received (NSAID, surgery, or rehab). The third part included a validated Swedish translation of the American College of Veterinary Surgeons (ACVS) COI (27).

The COI is a survey that provides a response scale from 1 to 5 for 16 questions, which are grouped into four categories or indices: *stiffness, function, lameness*, and *quality of life*. For each category, a standardized score was calculated by dividing the raw score by the maximum achievable score (Table 2).

**Table 2:** Part C in the questionnaire based on the validated ACVS COI, modified and validated in Swedish after translation.

**Group and item**	**Minimum and maximum values (raw score)**	**Range of the standardized score for each group**
**STIFFNESS (STF)**	**5–25**	**0.2–1**
(1) How severe is your dog's stiffness after first wakening in the morning?	1–5	-
(2) Later in the day, how severe is your dog's stiffness after lying down for at least 15 min?	1–5	-
(3) How much of a problem does your dog have rising to standing after lying down for at least 15 min?	1–5	-
(4) In general, over the past month, how much difficulty has your dog had with his or her joints?	1–5	-
(5) How often did your dog 'pay' for over-activity, with increased pain or stiffness the following day?	1–5	-
**FUNCTION (FN)**	**4–20**	**0.2–1**
(6) Jumping up (as in getting into the car or onto the bed)?	1–5	-
(7) Jumping down (as in getting out of the car or off of the bed)?	1–5	-
(8) Climbing up (as in stairs, ramps or curbs)?	1–5	-
(9) Climbing down (as in stairs, ramps or curbs)?	1–5	-
**LAMENESS/GAIT (LMS)**	**4–20**	**0.2–1**
(10) On average, how severe was your dog's limp during mild activities (such as short walks)?	1–5	-
(11) On average, how severe was your dog's limp during moderate activities (such as long walks, playing or running)?	1–5	-
(12) How often did your dog limp the day after moderate activities (such as long walks, playing or running)?	1–5	-
(13) How often have you been aware of your dog's joint problems?	1–5	-
**Quality of life (QOL)**	**2–10**	**0.2–1**
(14) In the past 4 weeks, what has been your level of concern that your dog's joint problems will shorten his or her life?	1–5	-
(15) In the past 4 weeks, what has been your level of concern that your dog is generally slowing down?	1–5	-
**PERCEPTION (PER)**	**1–5**	**0.2–1**
(16) Overall, how would you rate your dog's quality of life over the past 4 weeks?	1–5	-

*The owner's opinion including the past month was requested. Individual questions were rated on a five-point scale, where 1 indicated no condition, and 5, very severe condition*.

### Statistical Analysis

Descriptive statistics and chi-square testing were used for exploratory analyses and to identify potential unbalanced sampling. The univariate associations between ED grade and demographic-, lameness-, treatment-, and COI-related variables were assessed by means of multinomial ordinal logistic regression (significant at *p* < 0.05). The COI has been analyzed for internal correlations, and its composition was adjusted accordingly, including separating item 16 into a *perception score* (27).

To account for the complex, inter-related variables that affect the quality of life of dogs, we used additive Bayesian network modeling to identify the direct and indirect associations between our variables of interest (Table 3): ED grade (0–2), dog demographics, owner-reported lameness, type of treatment received, and COI index scores (LMS = lameness, STF = stiffness, FNC = function, QOL = quality of life, PER = owner's perception). The model included dogs with complete data for all. Additive Bayesian network modeling is a multivariate analysis method that uses a machine learning algorithm to determine the optimal statistical model directly from observed data, allowing all variables to be potentially response and explanatory (28). A detailed description of the additive Bayesian network model used in this study can be found in the **Appendix I**. All the analyses have been carried out using the software R (29) and JAGS (30).

**Table 3:** Variables included in the additive Bayesian network model.

**Variables included in the model**	**Abbreviation and color coding**	**Outcome vs. reference class**
American Staffordshire breed	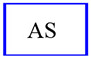	AS vs. other breeds
Bernese mountain dog breed	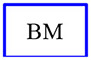	BM vs. other breeds
German Shepherd breed	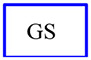	GS vs. other breeds
Labrador Retriever breed	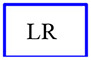	LR vs. other breeds
Rottweiler breed	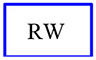	RW vs. other breeds
Elbow dysplasia grade 1	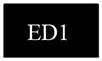	ED1 vs. ED0
Elbow dysplasia grade 2	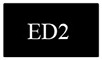	ED2 vs. ED0
Dog being neutered	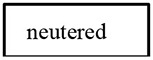	Neutered vs. not neutered
Dog treated with NSAID for elbow	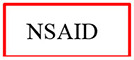	NSAID vs. no NSAID
Dog went to rehab for elbow	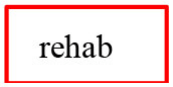	Rehab vs. no rehab
Dog received surgery for elbow	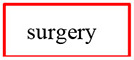	Surgery vs. no surgery
Lameness score	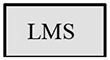	Poor LMS vs. good LMS
Stiffness score	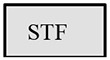	Poor STF vs. good STF
Function score	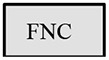	Poor FNC vs. good FNC
Quality of life score	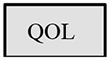	Poor QOL vs. good QOL
Owner's perception score	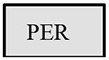	Poor PER vs. good PER
Gender of dog	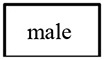	Male vs. female
Lameness in the last month	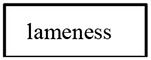	Lameness vs.no lameness
Age of dog at interview	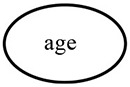	Years (continuous variable)

## Results

### Descriptive Statistics

Data were collected for 765 dogs, whose owners were interviewed between January and December 2017. There was a significant statistical association between the occurrence of euthanasia due to orthopedic disease and ED scores of 1 and 2 in the sample (chi-square *p* < 0.001). Sixty-one dogs were euthanized for orthopedic reasons and therefore excluded from both the orthopedic index part of the study and the statistical analyses.

The remaining 704 dogs (323 males and 381 females) had a median age at the time of interview of 5 years (min = 3, max = 10). One-fourth were castrated (*n* = 190, 27%). The distribution of dogs across ED status was slightly below the target number of 300 (Table 4).

**Table 4:** Overview of demographic and treatment variables by elbow dysplasia (ED) score.

	**ED0**	**ED1**	**ED2**	**Total**	**Proportion**
Overall number of dogs	269	238	197	704	100%
**Breed**					
American Staffordshire terrier	35	34	26	95	13%
Bernese mountain dog	56	55	36	147	21%
German Shepherd	53	40	46	139	20%
Labrador Retriever	67	55	52	174	25%
Rottweiler	58	54	37	149	21%
**Gender**					
Female	159	131	91	381	54%
Male	110	107	106	323	46%
**Neutering**					
No	210	168	136	514	73%
Yes	59	70	61	190	27%
**Age (years)**					
Median	5	6	6	5	-
Mean	5.37	5.65	5.72	5.56	-
Standard deviation	1.38	1.23	1.46	1.36	-
Range (min–max)	3–10	3–9	3–10	3–10	-
**Lameness in the past month**					
No	227	198	138	563	80%
1 leg	33	30	41	104	15%
>1 leg	9	10	18	37	5%
**Sought veterinary care**					
No	201	147	113	461	65%
Yes	68	91	84	243	35%
**NSAID treatment**					
No	29	39	22	90	13%
Yes	9	41	57	107	15%
Not applicable	230	136	118	484	69%
*Not available*	1	22	0	23	3%
**Elbow surgery**					
No	33	55	55	143	20%
Yes	4	17	22	43	6%
Not applicable	232	166	120	518	74%
**Rehab treatment**					
No rehab	18	39	44	101	14%
Other rehab	16	14	10	40	6%
Elbow rehab	4	26	31	61	9%
Not applicable	231	159	112	502	71%
**Stiffness score (STF)**					
Good (index = 0.2)	203	166	104	473	67%
Poor (index > 0.2)	66	72	93	231	33%
**Function score (FNC)**					
Good (index = 0.2)	254	204	151	609	87%
Poor (index > 0.2)	13	28	42	83	12%
*Not available*	2	6	4	12	1%
**Lameness score (LMS)**					
Good (index = 0.2)	230	176	111	517	73%
Poor (index > 0.2)	39	59	86	184	26%
*Not available*	0	3	0	3	<1%
**Quality-of-life score (QOL)**					
Good (index = 0.2)	236	179	117	532	76%
Poor (index > 0.2)	33	56	79	168	24%
*Not available*	0	3	1	4	1%
**Owner's perception score (PER)**					
Good (index = 0.2)	183	154	127	464	66%
Poor (index > 0.2)	46	50	49	145	21%
*Not available*	40	34	21	95	13%

*Values refer to the number of dogs per category, unless otherwise stated*.

Eighty percent of the investigated dogs (*n* = 563) had no owner-reported lameness in the past month, 15% displayed lameness on one leg (*n* = 107), and the remaining 5% were lame on more than one leg (*n* = 37). There was a significantly higher frequency of lameness among ED2 dogs compared to ED0 and ED1 dogs (*p* = 0.005).

One-third of the interviewed owners (*n* = 243) reported having sought veterinary care for their dogs for orthopedic reasons at any time of the dog's life. Of these dogs, 107 (44%) had been given NSAID treatment for the elbow at least once, 61 (25%) underwent elbow rehabilitation, and 43 (18%) underwent elbow surgery. The surgically treated dogs mainly had arthroscopy as the sole method of intervention (79%). Two dogs were treated with proximal abducting ulna osteotomy (4%), and in seven (16%) cases, the owners were not aware of the surgical method used. From a preliminary univariable analysis, higher ED grade showed a crude positive association with seeking veterinary care (*p* < 0.001), NSAID treatment (*p* < 0.001), and rehabilitation (*p* = 0.001) but not with elbow surgery (*p* = 0.11).

The outcomes of the COI were skewed toward lower (i.e., better) values. For all five indices (including PER), the median value of the standardized score was 0.2 (which was the lowest possible value, i.e., the most favorable outcome). For FNC, QOL, and PER, low values (0.2) were present in 75% of the responses. Given these very skewed score distributions, we decided to dichotomize the standardized scores for the multivariate analysis, using a conservative cut-off. Values =0.2 were considered “good,” whereas values >0.2 were considered “poor.” The dichotomous COI scores by ED grade are presented in Table 4.

### Multivariate Analysis

**Figure 1:**
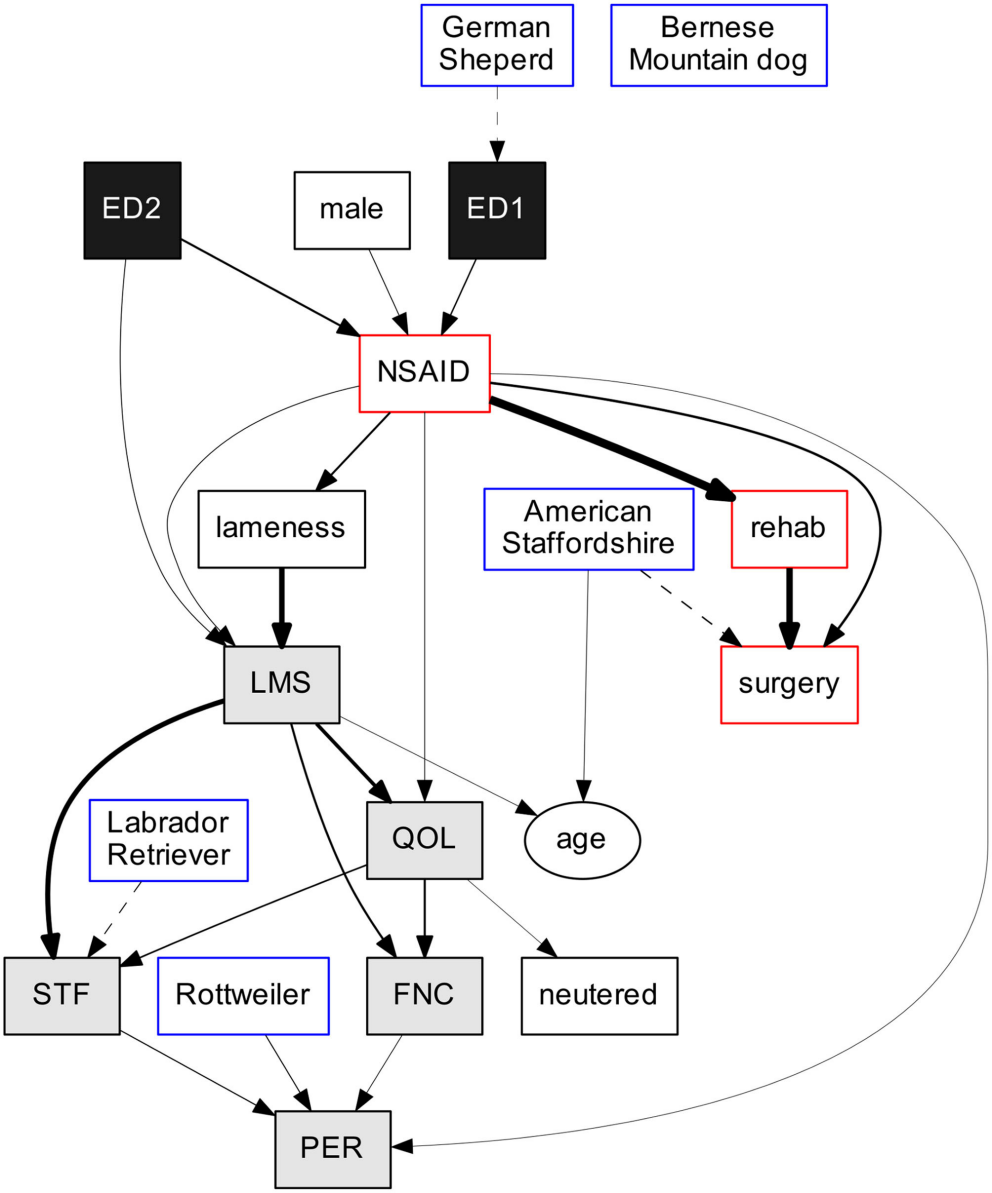
Outcome of additive Bayesian network model. Rectangles indicate binary variables, and ovals, continuous ones. Solid arrows indicate positive association, while dashed arrows, negative association. Arrow thickness reflects the link strength. Variables are color-coded according to their classification: black for elbow dysplasia (ED) grade, gray for Canine Orthopedic Index (COI), white for demography and lameness, red-contoured for treatment type, and blue-contoured for breed.

The output of the additive Bayesian network modeling is outlined graphically in Figure 1. This directed acyclic graph summarizes the factorization of the joint probability distribution of all variables included in the model. Each box represents a random variable, while arrows represent probabilistic dependencies between them. Solid arrows represent a positive association, and dashed arrows, a negative association. The thickness of the arrows reflects the strength of the association between two connected variables and is also quantified as link strength percentage (LS%) in Table 5. The direction of the arrows represents the flow of the predictive information. Incoming arrows to a node and regression coefficients (Table 5) encode the way the index node is predicted based on its parent set (i.e., which distribution to use to model the error term).

**Table 5:** Estimates of the marginal posterior densities (median and 95% credible interval) and link strength percentage (LS%) for the variables included in the model.

Links^*^	**Marginal value**	**95% credible interval**	**Interpretation**	**LS%**
STF ← LMS	17.2	[10.3–29.5]	Odds ratio	23.8%
STF ← QOL	4.4	[2.6–7.6]	Odds ratio	7.7%
STF ← LR	0.39	[0.22–0.67]	Odds ratio	4.9%
FNC ← LMS	7.1	[3.7–14.2]	Odds ratio	10.3%
FNC ← QOL	6.5	[3.4–12.8]	Odds ratio	10.0%
LMS ← ED2	3.0	[1.8–5.0]	Odds ratio	4.1%
LMS ← NSAID	3.7	[2.0–6.8]	Odds ratio	4.0%
LMS ← lameness	23.9	[13.8–43.1]	Odds ratio	25.6%
QOL ← LMS	9.6	[6.2–15.3]	Odds ratio	16.9%
QOL ← NSAID	3.4	[1.97–5.8]	Odds ratio	3.7%
PER ← STF	3.3	[2.1–5.3]	Odds ratio	5.3%
PER ← FNC	2.8	[1.6–5.0]	Odds ratio	3.5%
PER ← RW	3.1	[1.9–5.1]	Odds ratio	4.9%
PER ← NSAID	2.4	[1.4–4.0]	Odds ratio	2.7%
ED1 ← GS	0.29	[0.15–0.53]	Odds ratio	2.6%
Surgery ← AS	0.05	[0.0–0.35]	Odds ratio	6.9%
Surgery ← rehab	36.1	[12.6–114.5]	Odds ratio	29.8%
Surgery ← NSAID	11.2	[3.5–40.0]	Odds ratio	11.5%
Rehab ← NSAID	54.5	[25.4–131.5]	Odds ratio	39.6%
NSAID ← ED1	7.0	[3.4–16.0]	Odds ratio	6.7%
NSAID ← ED2	10.4	[5.1–23.6]	Odds ratio	10.0%
NSAID ← male	2.7	[1.7–4.4]	Odds ratio	4.2%
Lameness ← NSAID	6.1	[3.8–9.8]	Odds ratio	9.5%
Neutered ← QOL	2.3	[1.6–3.5]	Odds ratio	2.6%
Age ← LMS	0.46	[0.29–0.63]	Correlation	2.9%
Age ← AS	0.56	[0.33–0.79]	Correlation	3.3%

* *Variables are named so that the one at the left-hand side of the arrow is the outcome and the one at the right-hand side is the predictor. For instance, “surgery ← NSAID” reads as likelihood of surgery treatment given NSAID treatment and represents the odds ratio of receiving elbow surgery in case NSAID treatment was administered, in comparison to no NSAID treatment. In this case, NSAID-treated dogs were 11.2 more likely to have had received surgery compared to non-NSAID-treated dogs*.

#### ED Grade

ED1 and ED2 were positively directly associated (i.e., solid arrow) with NSAID treatment for elbow pain, meaning that dogs in both ED1 and ED2 groups were more likely to receive NSAID treatment compared to ED0 dogs. In fact, the odds ratio for NSAID treatment was 7.0 for ED1 dogs compared to ED0 dogs (Table 5, NSAID ← ED1 = 7.0) and 10.4 for ED2 dogs compared to ED0 dogs. ED2 grade was also positively associated with lameness score (LMS), with ED2 dogs three times more likely to have a poor lameness score (i.e., >0.2, Table 4) compared to ED0 dogs (LMS ← ED2 = 3.0) (Table 5).

#### Lameness

As expected, owner-reported lameness was directly associated with COI lameness score as well as with NSAID treatment; dogs treated with NSAIDs were more likely than untreated dogs to have experienced lameness in the past month.

#### Treatment Options

NSAID treatment showed the most complex relationship with other variables in the model. It was directly linked to nine other variables, both as a predictor and as an outcome. It was associated with both rehab and surgery, suggesting a close relation among treatment options. It seems that dogs receiving NSAID treatment were more likely to also receive rehab and/or surgery compared to untreated dogs. Unfortunately, due to the cross-sectional nature of the study, we could not determine whether there was a sequential use of treatment options.

Rehabilitation was strongly positively associated with surgery (LS = 29.8%) and with NSAID treatment (LS = 39.6%).

#### COI Scoring

The five orthopedic indices were highly inter-correlated, as well as associated with NSAID treatment and some breeds. Labrador retrievers had a better STF score than other breeds (odds ratio for STF ← LR = 0.34), while Rottweilers had a worse PER score than other breeds (odds ratio for PER ← RW = 3.1).

LMS was positively directly associated with STF, FNC, and QOL, meaning that dogs with a poor LMS score were more likely to score poorly on STF, FNC, and QOL as well. LMS score was positively associated with age, indicating that dogs with a poor LMS score were older. Interestingly, QOL was not directly linked to PER.

## Discussion

The aim of this study was to provide a better understanding of the predictive value of ED screening for quality of life in dogs not primarily seeking veterinary care for lameness. Our first hypothesis was that ED1 and ED2 dogs had worse owner-assessed quality of life compared to ED0 dogs. This was not statistically confirmed, and therefore, the first part of our hypothesis was rejected. The second part of the hypothesis, that the ED-positive dogs (ED1 or ED2) would suffer more from lameness and receive more veterinary care compared to the ED0 dogs, was confirmed in this study. These results can be used to increase owner awareness regarding their dogs' ED screening result. They can also be used to educate the owners about expected future elbow function of their young dogs as well as the possibility of a good quality of life despite an ED1 or ED2 result on elbow screening.

The COI indices were all linked together through several pathways (Figure 1, gray-shaded boxes), which was expected, as they represent different aspects of a dog's quality of life. Strong links were seen between LMS and STF as well as LMS and QOL. It is not surprising that lame dogs (due to OA) also show signs of stiffness after rest and that lameness also affects the owner-perceived quality of life (13). COI indices also showed direct and indirect connections with virtually all other variables in the model, suggesting that quality of life is driven by multiple factors. It was interesting to find that the standardized index for quality of life (QOL) was not directly linked to the owner's perception of quality of life (PER), suggesting that subjectivity and an emotional bond play a role when self-assessing the well-being of a pet. The owner–dog relationship may affect the owner's perception of the dog's quality of life (31).

Treatment variables were all linked together (Figure 1, red-contoured boxes), but only NSAID treatment showed direct connections with COI parameters.

NSAID treatment was directly associated with worse LMS, QOL, and PER scores. NSAID treatment was also indirectly associated with worse STF and FNC scores. An explanation for such a connection between NSAID treatment and worse COI indices may have to do with the owner's awareness and perception of the disease and his/her previous intention to treat. Another possibility for this finding is the likelihood of NSAIDs being the first and, many times, only treatment provided in dogs with clinical symptoms of ED. This could have an impact on the outcome scores, and the finding may be an artifact of the inability to account for simultaneous treatments.

On the other hand, rehabilitation and surgery did not show any direct association with COI indices, suggesting that dogs receiving rehab and/or surgery were not perceived as having a better or worse quality of life than their untreated counterparts.

Interestingly, we found that ED grade was not directly linked to COI indices, except for ED2 and lameness score (LMS). This means that dogs in the ED2 group were perceived by their owners as being more impaired by lameness than dogs in the ED0 and ED1 groups. For all the other COI indices (function, stiffness, quality of life, and owner's perception), there was no direct association with ED grading. The lack of associations may be caused by the skewed distribution of the COI indices, meaning that the lack of variability in scores weakened any potential associations. The crude association between ED scoring and COI indices identified in the preliminary univariate analysis was revealed to be only an indirect association, emphasizing the importance of a multivariable approach in the analysis.

Dogs with ED2 had moderate OA at the time of radiography, yet only 43% received veterinary care for orthopedic reasons at any point during the following 2- to 6-year period. In most cases, this is probably due to owner perception that no lameness/stiffness is observed that needs veterinary attention, but it could also be argued that some dogs with ED2 simply do not show clinical signs of OA until later in life. Another reason for this finding may be that owners are expecting some degree of lameness in their ED2 dog and therefore do not seek veterinary care. Irrespective of cause, this observation is important since the owners are the ones who decide to seek veterinary counseling and who evaluate the quality of life of their pets on a day-to-day basis.

Breed differences were found. The most important were: (1) German Shepherds were less likely to have ED1 grading compared to the other breeds. (2) Labrador Retrievers were less likely to be perceived by their owner as stiff compared to the other breeds with the same ED grade. (3) Rottweilers were more likely to have a higher PER score, meaning that owners of Rottweilers experience their dogs as suffering more than the other breeds with the same ED score in this study. These results may be speculated to be due to the breed's different mental and physical status as well as the owner's perception of the breed.

There are several limitations to this study. One is that the only outcome measurement is based on the owners' perception of their dogs' quality of life and therefore may be subject to recall bias. It is possible that when asked questions over the telephone, owners could have misremembered their dog's medical history, possibly underestimating the severity of its ED symptoms. Owners declining participation in the interview may represent another source of bias, as owners with the worst experience may have been less likely to consent. In addition, given the cross-sectional nature of the study, the variables under investigation had different temporal references: ED screening was performed when the dog was around 12 months old; treatments could have happened at any time prior to the interview; lameness was reported for the month prior to the interview, and the COI indices referred to the time of the interview. This did not allow us to make any inference about the causality in the identified associations, as we did not have enough information on the chain of events to build a causal diagram. The results are limited by an inability to differentiate between the timings of the reported treatments.

There is some evidence that owners may not be able to accurately evaluate lameness in dogs with ED (32), such as the study by Walton et al., which found a weak correlation between OA questionnaires and objective measurements (33). Additionally, the owners are not trained in grading lameness and may be biased by knowing their dog's ED grade.

Another limitation is the potentially inaccurate assessment of ED, due to it being based on a single flexed lateral projection of the elbow. This could have led to ED being missed (34), since OA may not have yet developed at the time of radiography (35, 36), or overestimation of the frequency of ED when based solely on radiographic score, since the Kennel Club score could be detecting both conditions that are developmental and those that are acquired in origin. A stronger study would have been to perform an orthopedic clinical examination, COI, and objective gait analysis at the time of initial radiographic evaluation and then recall all subjects for updated clinical evaluation (radiographs, orthopedic evaluation COI, and objective gait analysis).

Simultaneous treatment with medications other than NSAIDs such as paracetamol, gabapentin, tramadol, as well as other treatments was asked for during the interview, but since the frequency of simultaneous treatment options was extremely low overall, no further analysis of these results was performed.

## Conclusion

It is of paramount importance for the surgeon to decide what criteria to use prior to surgical intervention. Should surgery be performed in dogs based solely on radiographic mild to moderate confirmed OA with minor clinical signs? In most surgically treated animals in this study, an elbow arthroscopy alone was the only reported surgical intervention; however, arthroscopic intervention may not show benefits over conservative treatment (37).

In this study, information on the effect of mild to moderate ED on quality of life in five different breeds is presented. This is a first step to increase our understanding on how ED grades from a screening program are related to the long-term well-being of dogs from the owner's perspective. The results showed that ED1- and ED2-graded dogs were much more likely to receive treatment than ED0-graded dogs. However, there was no direct association between dog's quality of life, as perceived by the owner, and ED grading, except for lameness score and ED2. These results can be used to increase owner awareness regarding the ED screening results of their dogs. The output of this study can be used to educate the owners about expected future elbow function of their young dogs as well as the possibility of a good quality of life despite an ED1 or ED2 result on elbow screening.

## Data Availability Statement

The raw data supporting the conclusions of this article will be made available by the authors, without undue reservation.

## Author Contributions

AB and ML initiated the study. SJ was involved in collecting the data. AC conducted the data analysis and interpretation of the results. All authors participated in the manuscript preparation, discussions, revisions of the text, read, and approved the final manuscript.

## Conflict of Interest

The authors declare that the research was conducted in the absence of any commercial or financial relationships that could be construed as a potential conflict of interest.
