# Supplementary material for: Long-Term Prognosis of Quality of Life in Dogs Diagnosed With Mild to Moderate Elbow Dysplasia in Sweden
Source: Front Vet Sci. 2020 Nov 3;7:572691. doi: 10.3389/fvets.2020.572691 (PMC7669830; doi:10.3389/fvets.2020.572691)
Supplement: Supplementary file 1 [file Data_Sheet_1.PDF]

Additive Bayesian Network (ABN) model

The modelling was carried out using the open source software R (1) and JAGS (2), and involved three consecutive steps: (i) identifying a globally optimal DAG, (iii) adjusting the chosen model for over-fitting, and (iii) estimating parameters.

I. IDENTIFICATION OF THE GLOBALLY OPTIMAL MODEL

In ABN modelling, finding the model with best goodness of fit to the available data is known as structure discovery. This is usually performed by selecting the model with the highest network score. The network score represents the marginal log likelihood of the data given the model (3) and it includes an implicit penalty for model complexity. The network score is decomposable, which means that it can be computed separately for each node, making its computation very efficient.

In our study, we used the R package *abn* (version 1.0.2) (4) for structure discovery, which computes the network score using Laplace approximation at each node (5). The identification of the maximum a posteriori ABN (i.e. the model with the highest overall network score) was achieved with an order-based exact search method (6), which identifies the ABN with the network score equal to the best possible network score of any ABN. Given that the computational cost of identifying the best fitting ABN increases super-exponentially with the network complexity (i.e. the number of covariates – or parents – at each node), the model search was iterated across incremental parent limits. This means that the model selection procedure started from one allowed parent per node and then the parent limit was increased, step by step, until the highest score was achieved, and the resulting network had fewer parents than the maximum number allowed. That equals to finding the minimal complexity needed to achieve the highest possible network score.

The model applied in this study uses a Bayesian approach for both structure discovery and parameter learning, and as such it relies on prior information. With respect to the model structure, a uniform prior distribution was chosen, assuming that all eligible network structures were equally plausible, to allow a fully data-driven approach. In term of parameter priors, we assumed weakly informative Gaussian priors with mean zero and variance 1000 for each of the regression parameters of the model, as well as diffuse Gamma priors (with shape and scale equal to 0.001) for the precision parameters in Gaussian node in the model.

Additional knowledge about data structure, that could guide the search for the optimal model, was included by banning some specific arcs from being considered in the final DAG. This was done by providing a *ban matrix* (Figure 1), where rows and columns represent children and parents, respectively, and 1 and 0 indicates whether the arc is banned or allowed. For example, all the arcs going to the variable “male” (i.e. third last row) were banned (i.e. all 1s) under the reasonable assumption that none of the considered variable was expected to influence the sex of the dog, which is an inborn trait. The information encoded in the ban matrix was subjectively chosen by the authors to reflect their belief about data structure, which in turn was derived from knowledge of the study design (e.g. the ED status was recorded before the COI questionnaire, therefore COI-related variables could not affect it) and thorough reasoning (e.g. it is reasonable to assume that inborn traits cannot be affected by any variable).

Figure 1: Ban matrix used in the model

	stf	fnc	lms	qol	per	AS	BM	LR	RW	GS	ED1	ED2	surgery	rehab	NSAID	lameness	male	neutered	age
**stf**		0	0	0	0	0	0	0	0	0	0	0	0	0	0	0	0	0	0
**fnc**	0		0	0	0	0	0	0	0	0	0	0	0	0	0	0	0	0	0
**lms**	0	0		0	0	0	0	0	0	0	0	0	0	0	0	0	0	0	0
**qol**	0	0	0		0	0	0	0	0	0	0	0	0	0	0	0	0	0	0
**per**	0	0	0	0		0	0	0	0	0	0	0	0	0	0	0	0	0	0
**AS**	1	1	1	1	1		1	1	1	1	1	1	1	1	1	1	1	1	1
**BM**	1	1	1	1	1	1		1	1	1	1	1	1	1	1	1	1	1	1
**LR**	1	1	1	1	1	1	1		1	1	1	1	1	1	1	1	1	1	1
**RW**	1	1	1	1	1	1	1	1		1	1	1	1	1	1	1	1	1	1
**GS**	1	1	1	1	1	1	1	1	1		1	1	1	1	1	1	1	1	1
**ED1**	1	1	1	1	1	0	0	0	0	0		1	1	1	1	1	0	0	1
**ED2**	1	1	1	1	1	0	0	0	0	0	1		1	1	1	1	0	0	1
**surgery**	0	0	0	0	0	0	0	0	0	0	0	0		0	0	0	0	0	0
**rehab**	0	0	0	0	0	0	0	0	0	0	0	0	0		0	0	0	0	0
**NSAID**	0	0	0	0	0	0	0	0	0	0	0	0	0	0		0	0	0	0
**lameness**	1	1	1	1	1	0	0	0	0	0	0	0	0	0	0		0	0	0
**male**	1	1	1	1	1	1	1	1	1	1	1	1	1	1	1	1		1	1
**neutered**	0	0	0	0	0	0	0	0	0	0	0	0	0	0	0	0	0		0
**age**	0	0	0	0	0	0	0	0	0	0	0	0	0	0	0	0	0	0	

II. ADJUSTMENT FOR OVERFITTING

ABN modelling is known to be prone to overfitting (7). Therefore, a parametric bootstrapping approach using Markov chain Monte Carlo (MCMC) simulations was used to address this issue, as described in Lewis and McCormick (8). Briefly, the model chosen from the exact search (outcome of step ii) was used to generate 5000 bootstrap datasets of equal size to the original dataset. These simulations were computed using JAGS and the R package *rjags* (version 4-8). Each bootstrap dataset was then treated as if it were the original data, and a globally optimal DAG was identified exactly as described before (i.e. exact search with incremental parent limit). This bootstrapping process generated 5000 different DAGs. To address over-fitting, any arcs in the DAG from the original data which were not recovered in >50% of the bootstrap DAGs were deemed to have insufficient statistical support to be considered robust (3). These arcs were therefore removed, obtaining a final pruned DAG, equivalent to a multivariate GLM.

III. PARAMETERS ESTIMATION

The marginal posterior log odds ratio and 95% credible intervals were estimated for each parameter from the posterior distribution, expressed by the DAG identified at the second step. Being in a Bayesian statistics framework, the parameters were the maximum likelihood estimates (MLE) based on the joint posterior distribution. With ABN methodology, it is possible to evaluate the association between all variables, including the outcome and hence evaluate all relationships present in the data. An arc between two variables in the final ABN model is referred to as a “direct” relationship, whereas an “indirect” relationship is defined as two arcs connecting two variables with an intermediate variable. In order to estimate the parameters of the linked variables, a specific function (fitabn) of the R package *abn* was used.

REFERENCES

1. R Core Team. R: A language and environment for statistical computing. Vienna, Austria.: R Foundation for Statistical Computing; 2017.
2. Plummer M, editor JAGS: A program for analysis of Bayesian graphical models using Gibbs sampling. Proceedings of the 3rd international workshop on distributed statistical computing; 2003: Vienna, Austria.
3. Lewis FI, Brulisauer F, Gunn GJ. Structure discovery in Bayesian networks: an analytical tool for analysing complex animal health data. Preventive veterinary medicine. 2011;100(2):109-15.
4. Kratzer G. PM, Lewis F.I., Furrer R. abn: an R package for modelling multivariate data using additive Bayesian networks. [R Vignette]. In press 2016.
5. Tierney L, Kadane JB. Accurate approximations for posterior moments and marginal densities. Journal of the american statistical association. 1986;81(393):82-6.
6. Koivisto M, Sood K. Exact Bayesian structure discovery in Bayesian networks. Journal of Machine Learning Research. 2004;5(May):549-73.
7. Babyak MA. What you see may not be what you get: a brief, nontechnical introduction to overfitting in regression-type models. Psychosomatic medicine. 2004;66(3):411-21.
8. Lewis FI, McCormick BJ. Revealing the complexity of health determinants in resource-poor settings. American journal of epidemiology. 2012;176(11):1051-9.
